# Supplementary material for: Kinetic analysis of multistep USP7 mechanism shows critical role for target protein in activity
Source: Nat Commun. 2019 Jan 16;10:231. doi: 10.1038/s41467-018-08231-5 (PMC6335408; doi:10.1038/s41467-018-08231-5)
Supplement: Supplementary file 1 — Supplementary Information [file 41467_2018_8231_MOESM1_ESM.pdf]

Kinetic analysis of multistep USP7 mechanism shows critical role for target protein in activity

Kim et. al. 2018

Supplementary Information

Supplementary methods

NMR assignment 2

Chemical synthesis 2

Fmoc solid phase peptide synthesis 2

p53Ub and $p53_{short}Ub$ 2

$p53Ub_{inh}$ and $p53_{short}Ub_{inh}$ 3

$p53Ub_{VA}$ and $p53_{short}Ub_{VA}$ 3

^{TAMRA}p53 peptide 3

Global modelling of enzymatic activity 4

KinTek observables definition and data treatment 4

Reaction assumptions and rate determination 5

Remarks on USP7 CD 5

Remarks on USP7 TCD 5

Remarks on USP7 CD12345 6

Remarks on USP7 FL and statistical analysis 6

Supplementary figures 7

Supplementary figure 1. Related to Figure 2; 7

Supplementary figure 2. Related to Figure 2; 9

Supplementary figure 3. Related to Figure 3 and Figure 4d; 11

Supplementary figure 4. Related to Figure 5 and Figure 6b; 13

Supplementary figure 5. Related to Figure 6; 15

Supplementary figure 6. Related to Figure 6; 17

Supplementary figure 7. Related to Figure 6; 19

Supplementary figure 8. Related to Figure 6 and Supplementary figure 5; 21

Supplementary references 23

Supplementary methods

NMR assignment

To characterize the weak interaction between USP7-Ubl45 and USP7CD *in trans* we used solution NMR spectroscopy to map the binding interface at the residue-level. We isotope-labelled the smallest of the two domains, Ubl45, including the C-terminal peptide, and first assessed the quality of its NMR spectrum. The ^{1}H-^{15}N correlation spectrum of the 25 kDa Ubl45 domain, spanning from residue 889 until 1102, shows the dispersion and number of peaks that is expected for a well-folded domain of this size (Figure 2a). We could confidently assign resonances for 84% of the residues present in the construct (Supplementary Figure 1a). The assigned backbone chemical shifts were used to predict the secondary structure using the TALOS-N webserver [1]: the identified secondary structure matched the crystal structure of Ubl45 (PDB: 5JTV) very well (Supplementary Figure 1b).

Next, we titrated in the unlabelled interaction partner CD and followed the chemical shift perturbations (CSPs) for the assigned Ubl45 residues. The titration was performed using Ubl45:CD ratios of 1:1, 1:5 and 1:10 where the Ubl45 concentration is kept constant at 45 µM and the highest concentration of CD is 450 µM. Binding is apparent from the overall loss in peak intensities for residues in the folded core of the protein (Figure 2c). Due to the large size of the complex (60 kDa) resonances of the bound state are severely broadened. This is not the case for the tails of Ubl45, indicating that they remain flexible.

The tails did show the same uniform broadening however when titrating in covalently linked CDUb (Figure 2e). Again we kept the concentration of Ubl45 constant at 45 µM, titrating in CDUb in an 0.1 and 0.3 ratio, as the determined affinity is in the low micromolar range (Figure 3). The resulting intensity plot for the Ubl45 residues show a decrease correlated to the fraction of CDUb titrated in. Furthermore, we can now see that, unlike the CD only titration, the intensities belonging to both termini decrease, uniformly with the core domain. This could be due to the overall tighter binding, restraining the flexible termini, or to the interaction of the termini with the CDUb.

Chemical synthesis

Fmoc solid phase peptide synthesis

Peptides were synthesized on a Syro II MultiSyntech Automated Peptide synthesizer by standard 9-fluorenylmethoxycarbonyl (Fmoc) based solid phase peptide chemistry on a 25 or 50 µmol scale. Starting with the pre-loaded Fmoc amino acid resin (Applied Biosystems), each successive amino acid (Novabiochem) was coupled in 4 molar excess for 45 min with PyBOP and DiPEA. Deprotection of the Fmoc group was achieved with 20% piperidine in NMP (2×2 and 1×5 min). Peptides were cleaved with TFA/H_2O/phenol/*i*Pr_3SiH (90.5/5/2.5/2 v/v/v/v) for 3 hrs., precipitated in cold *n*-hexane/diethyl ether and washed 3× with diethylether. The pellet was dissolved in H_2O/CH_3CN/formic acid (65/35/10; v/v/v) and lyophilized. The purity of the peptides was determined by LC-MS analysis and peptides were purified by RP-HPLC where appropriate.

p53Ub and p53$_{short}$Ub

p53 peptides (residues 357-389 and 368-389) containing an thioLys at position 382 and a 5-carboxy-TAMRA at the N-terminus were synthesized according to the procedure described above. Ub was

coupled to the peptides by native chemical ligation: To 0.1 M sodium phosphate pH 8.0 were added: 5 mM $MgCl_2$ (from 0.2 M aq. stock), 150 mM NaCl (from 5 M stock), 50 mM MESNa (from 2 M stock), 5 mM ATP (from 0.5 M aq. stock), Ub (50 µM) and TAMRA-thiolysine modified p53 peptide (250 µM). After adjusting the pH to 7.5 – 8.0 using 1M NaOH, E1 enzyme (150 nM, UBE1, Boston Biochem) was added and the ligation mixture was incubated at 37°C for 6 hrs. Next, 20 mM VA-044 (from freshly prepared 0.1 M stock in buffer), 40 mM GSH and 0.25 M TCEP (0.5 M stock, pH 7.0) were added to the crude ligation mixture. The pH was corrected to 7.0 using 1N NaOH and the reaction mixture was incubated at 37°C for 6 hrs. The constructs were purified by RP-HPLC on a Shimadzu LC-20AD/T using a C8 Vydac column (Grace Davison Discovery Sciences™). Column mobile phases: A= 0.05% aq. TFA and B= 0.05% TFA in CH_3CN. T= 40°C. Flow rate= 5 mL min^{-1}. Gradient: 30→75%B over 18 min.

p53Ub$_{inh}$ and p53$_{short}$Ub$_{inh}$

p53 peptides (residues 357-389 and 368-389) containing an azido-ornithine at position 382 were synthesized according to the procedure described above. Ub was coupled to the peptides by the copper-catalyzed alkyne-azide cycloaddition (CuAAC or 'click') reaction: Ub-PA and the azido-modified p53 peptide were dissolved in warm DMSO at a concentration of 50 mg mL^{-1}. 1 eq. of the p53 peptide was mixed with 0.9 eq. of Ub-PA (typically 90 µL) in 1 mL 8M urea, 100 mM phosphate buffer pH 7. To the resulting solution 10 µL of catalyst solution containing 20 mg mL^{-1} Cu(I)Br in MeCN and 50 mg mL^{-1} TBTA-analogue in MeCN (2:3, v/v) was added followed by a short vortex, repeated in 5 minute intervals 5 times in total. After reactions were finished, as judged by LC-MS (~ 1 hr.), the reaction was quenched by the addition of 100 µL of 0.5 M EDTA, pH 7.0. The product was purified using RP-HPLC on a Waters Atlantis T3 C18 30x250 5µm. Column Mobile phases: A= 0.05% aq. TFA and B= 0.05% TFA in CH_3CN. Flow rate= 18 mL min^{-1}. Gradient: 20→32%B over 20 min.

p53Ub$_{VA}$ and p53$_{short}$Ub$_{VA}$

p53 peptides (residues 357-389 and 368-389) containing a Dab(Alloc) at position 382 were synthesized according to the procedure described above. The resin was treated with $Pd(PPh_3)_4$ (0.35 eq) and Ph_3SiH (20 eq) in DCM (2x 20 min) and shaken overnight at room temperature with 4-((*tert*-butoxycarbonyl)amino)-3-(*tert*-butyldisulfanyl)butanoic acid (3 eq), PyBOP (3 eq) and DiPEA (6 eq). After extensive washings (3× NMP, DCM and Et_2O) the resin was treated with TFA/H_2O/phenol/iPr_3SiH (90/5/2.5/2.5 v/v/v/v) for 3 hrs. followed by precipitation in cold Et_2O/pentane 3:1 v/v. The crude peptide was lyophilized and purified by HPLC. Ub_{75}SEt was coupled to the peptides by native chemical ligation: To a solution of the p53 Dab mutant in 0.15 M sodium phosphate buffer (pH 7) containing 6M Gdn·HCl and MPAA (250 mM), a solution of Ub_{75}SEt in 0.2 M sodium phosphate buffer (pH 7) containing 6M Gdn·HCl and MPAA (250 mM) was added and the mixture (conc.: 50 mg mL^{-1}) was incubated at 37°C overnight. Next, TCEP was added to reduce the MPAA disulfide. The product was purified using RP-HPLC on a Waters Atlantis T3 C18 30x250 5µm. Column Mobile phases: A= 0.05% aq. TFA and B= 0.05% TFA in CH_3CN. Flow rate= 18 mL min^{-1}. Gradient: 20→40%B over 25 min.

^{TAMRA}p53 peptide

p53 peptide (residues 357-389) with a 5-carboxy-TAMRA at the N-terminus was synthesized according to the procedure described above. LC-MS analysis was performed on a system equipped

with a Waters 2795 separation Module (Alliance HT), Waters 2996 Photodiode Array Detector (190−700 nm) and a Micromass LCT-TOF Premier mass spectrometer. Samples were run over an XBridge BEH300 C18 column (5 µm, 4.6×100mm, T= 40°C). Samples were run at 0.8 mL min^{-1} using a gradient of two mobile phases: A= 1% acetonitrile and 0.1% formic acid in water; B= 1% water and 0.1% formic acid in acetonitrile. Gradient 30−60%B over 6.5 min.

Global modelling of enzymatic activity

All the acquired data for USP7 activity on the model substrate p53Ub were loaded into KinTek [2] per construct. Here we describe the corrections applied to the data before introduction and how we defined our observables. We also state our assumptions and what steps were taken to condense the model in order to be dependent on the least amount of variables.

KinTek observables definition and data treatment

The minimal substrate data (panel A in Figure 6, S4, S5, S6) were obtained using an injector in our plate reader set up, so no time delay had to be introduced. The raw data could be converted to amounts (µM) using a calibration curve and then directly read into KinTek.

The FP data on the model substrate from the plate reader (panel b, upper) required a delay before introduction into KinTek. This delay was estimated for each experiment by co-fitting the curves using Prism 7 to determine point "0". The curves were corrected for a drift using the null experiment and subsequently normalised (panel b, lower). Fittable data (with good amplitudes) were then read into KinTek, omitting concentrations within two orders of magnitude to the substrate concentration due to their lack of signal at the early time points. We defined the observed curves as amount of uncleaved substrate (p53Ub) and the bound fraction of product (USP7.p53), although this is a minor influence, all scaled by a defined factor **c** of 1000 that converted the used 0.1 µM substrate to 100%.

In the stopped-flow FP activity anisotropy data (panel c) the raw data were read-in as is. For data quality purposes we excluded the early time points (<0.001 s) due to the low signal to noise ratio as well as the late time points (>10s), as we observed bleaching of the fluorophore. Here we scaled the observables using factor **a** determined from the null experiment as this curve represents the appropriate signal for the amounts in the reaction. We noticed how the curves, especially for full-length, represented four different phases (Figure 6c; description c) below), so we accounted for these with four different USP7 states each with their own scaling factor (FP1-4) that converged in the initial fitting of the experiment.

For the intensity signal accompanying the anisotropy data (Figure 6d), the overall signal differences were less than 10 percent (before the 10 s mark), allowing use of the obtained anisotropy signal without correction [3]. To use the intensity signal, we subtracted the baseline (null experiment) and normalised using the early time points (<0.005 s), setting these to an arbitrary 100. We then applied the same observables as for panel c, now scaling them with a defined factor **b** of 2000 (100 / 0.05 µM). We found that the intensity of the free substrate and product did not differ from their initial bound state (USP7.p53Ub and USP7.p53), but that there are two different USP7 states in the hydrolysis. These observations have been described with their own scaling factors (I1-I3).

For the constructs TCD and FL affinity for the p53 peptide product was high enough to be relevant (Figure 5c). For TCD, this value (2 µM) is far weaker than the 50 nM used in the stopped-flow experiments, so introduction of the fitted FP data from Figure 5c was sufficient input. For the full-

length construct however, the K_D (160 nM) was much closer to the used concentration, so we performed a separate stopped-flow experiment for the p53 product, to fit the data within the KinTek model. We pre-treated the anisotropy data similarly as in panel c, but now only for the product states, and a defined scaling factor **q** of 3.4 that related the used amount (25 nM) to the obtained signal in the null curve.
In panel f we used the intensity data belonging to the p53 product stopped-flow. The data are corrected just as in panel d, and the observables now only pertain to the product and its complexes. As the used concentration is two-fold lower (0.025 µM) the scaling factor **r** is now 4000.

```
a)   Rho
b)   c*(p53Ub+(USP7.p53+USP7*.p53))
c)   a*(p53Ub + FP1*(USP7.p53Ub) + FP2*(USP7*.p53Ub +
     USP7#.p53Ub) + FP3*p53 + FP4*(USP7.p53+USP7*.p53))
d)   b*((p53Ub + USP7.p53Ub) + I1*(USP7*.p53Ub) +
     I2*(USP7#.p53Ub) + I3*(p53+USP7.p53+USP7*.p53))
e)   q*(p53 + P1*(USP7.p53+USP7*.p53))
f)   r*(p53 + USP7.p53+(P2*USP7*.p53))
```

Reaction assumptions and rate determination
We fitted every experiment separately, to obtain good estimations for the scaling factors. We then determined the on-rate, which is diffusion-limited and should be shared over all experiments. To this end we used all stopped-flow anisotropy data, as these experiments have a direct indication of the association. We were able to pinpoint this rate at 179 µM s^{-1}, well within the range considered reasonable for diffusion [4]. Furthermore, we assumed an irreversible reaction, so only hydrolysis of the substrate (k_4), locking the reverse reaction (k_{-4}) at 0. For the model, we started out with as few steps as possible (binding, catalysis and release; Figure 6h), only adding intermediates when the data required them.

Remarks on USP7 CD
For CD we found that the amplitudes for the stopped-flow data were too small, making them unusable for KinTek fitting. Therefore, we used only the minimal substrate (Supplementary Figure 6a) and plate reader FP enzyme activity data (Supplementary Figure 6b) for fitting and included data from TCD for step 3 (Supplementary Figure 6e), assuming that the observed conformational changes are the same.

Remarks on USP7 TCD
The stopped-flow data for TCD indicated a delay phase (0.1-5 s), which we modelled as a conformational change step (*Step 2*), based on the intensity data (Supplementary Figure 4d). The data could be fitted with one state, which did not change shape, thus the observables description for c) could be condensed, merging the FP1 and FP2 terms. Furthermore, the FP4 term seemed to have a minor effect only after 10 s, prompting us to omit the term in the fit. Similarly, we condensed the terms for the accompanying intensity experiment. With the data we modelled both the minimal and the model substrate with the same set of reactions, linking the conformational change step (*Step 2*)

as well as the catalytic step (*Step 4*). Assuming a similar reaction for both substrates resulted in a good fit and values stated in Supplementary Figure 4g.

Remarks on USP7 CD12345

The data for CD12345 had low amplitudes, indicative of the low affinity for the substrate. This resulted in high sigma values in the modelling. Just as in the TCD case, we had to model one intermediate step for reasonable fits. Therefore we condensed the observable terms in a similar fashion as for TCD. Furthermore, as there is no detectable binding of p53 to the construct (Supplementary Figure 5c) we did not require the p53 product retaining line in the model.

When attempting to link the found values for the p53 substrate with the minimal substrate we found the catalytic rates non-comparable, possibly due to interaction with the p53 peptide. We therefore could not assume a similar catalytic rate and constraining the fits was not possible through this linking. Combined with the low amplitudes, the less constrained values were not converged enough to be assessed by the statistical analysis.

Remarks on USP7 FL and statistical analysis

In the fitting of the FL construct two intermediate steps, instead of one, were required. As both TCD and CD12345 needed one intermediate, the requirement for two in the FL construct added up. We modelled these steps as two separate distinct steps, assumed to be sequential. Furthermore, at the product release steps we had to add a conformational change step upon binding of the p53 product (*Step 5*) to satisfy the p53 peptide stopped-flow data (Figure 6f).

After fitting all the experimental data using the model description (Figure 6h), we performed the statistical analysis using the FitSpace module of KinTek [5] on the fitted data for TCD and FL (Supplementary Figure 7). The analysis showed us that some variables are heavily dependent on one another, but we could constrain their values by linking their ratios. Our data unfortunately do not have the resolution to pinpoint on- and off-rates for each step. The resulting values should therefore rather be interpreted as the optimal ratio to model the reaction or intermediate steps.

Supplementary figures

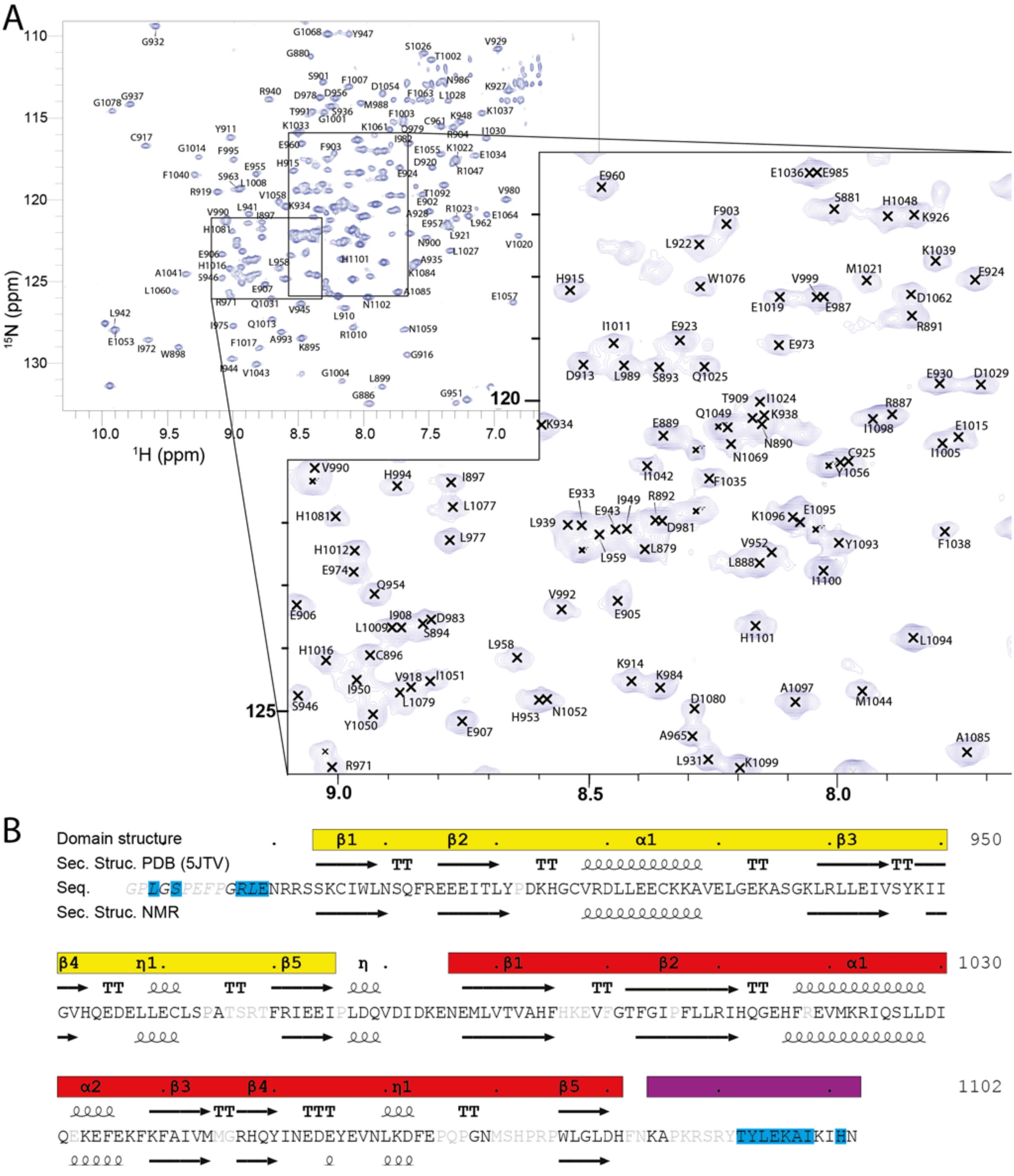

Supplementary figure 1. Related to Figure 2;

Supplementary figure 1. Related to Figure 2;

A. The ^{1}H-^{15}N correlation spectrum of Ubl45 with assignments indicated. The zoom shows the crowded central region. Smaller crosses are unassigned peaks.

B. Secondary structure of Ubl45 obtained from NMR backbone shifts. The coloured blocks on the first row represent the Ubl domains 4 (yellow) and 5 (red) and the C-terminal tail (purple). The secondary structure from the crystal structure, as determined by DSSP [6] for PDB: 5JTV, is depicted just below. The primary sequence is shown on the third row with tag residues in italics, assigned/unassigned residues in black/grey font and highly flexible residues shown with cyan background. The last row shows the secondary structure as determined from obtained chemical shifts (HN, N, Cα, Cβ, CO) using the TALOS-N webserver [1].

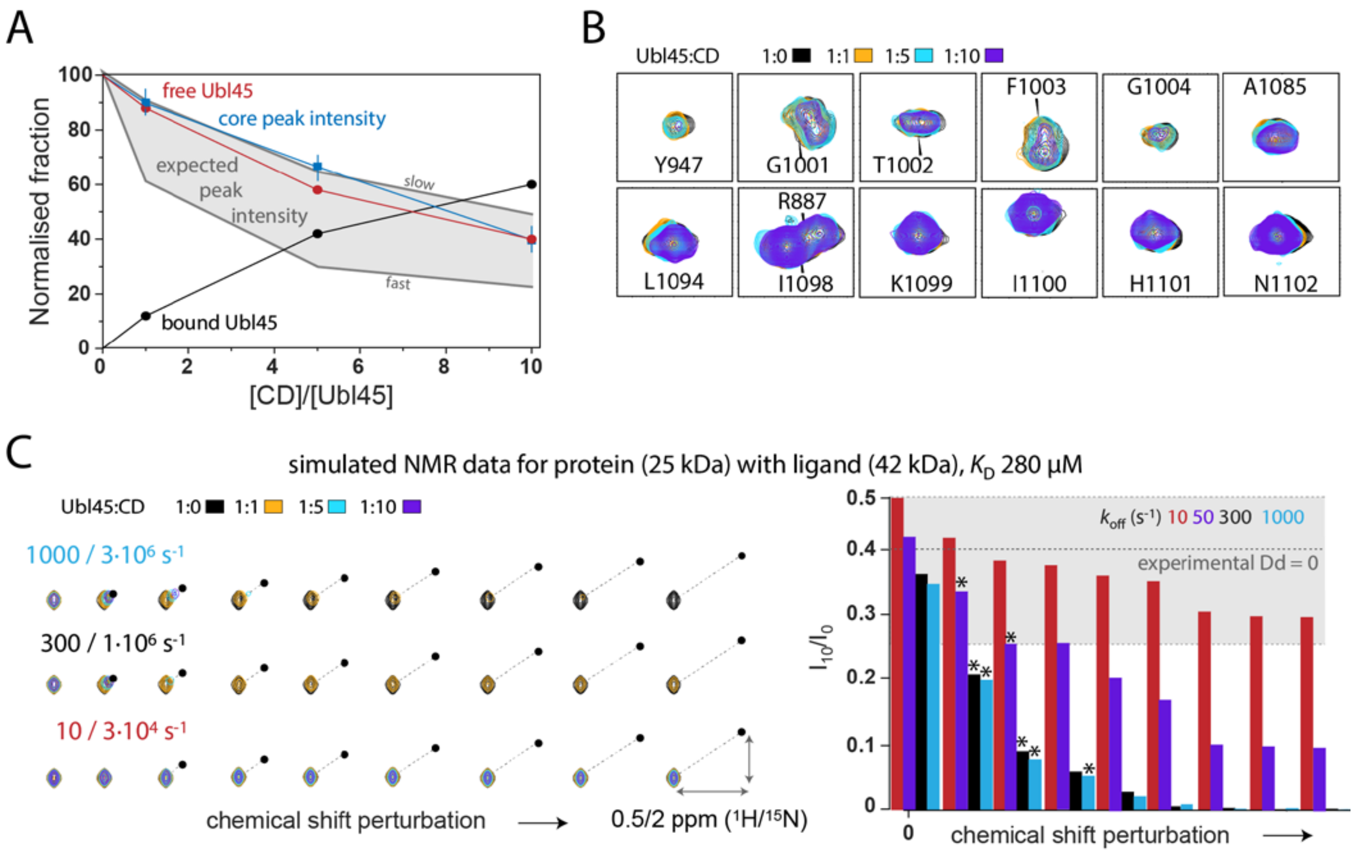

Supplementary figure 2. Related to Figure 2;

A. The overall peak intensity for Ubl45 residues in the folded core without chemical shift perturbations (blue) matches the expected values (grey) based on the affinity determined by SPR (Figure 3A, main text). The core peak intensity was derived from the average intensity over 93 residues (all with no or minimal chemical shift changes), excluding the 50% most intense peaks. For residues without chemical shift perturbations (CSPs), peak intensity is determined by both the population of free (red) and bound (black) states. Notably, because of the significantly larger size of the complex (67 kDa) compared to the free protein (25 kDa), signals from the bound state will have a ~2.5 times larger line width in each dimension [7,8] and will thus contribute less to the peak intensity. In addition, the dynamic exchange between free and bound state critically determines the observed peak shape and peak intensity, even for residues without chemical shift perturbation. In the limit of slow exchange, the signals of free and bound states add independently and a peak intensity of 50% of the initial value is expected at the end of the titration. In the limit of fast exchange, both free and bound states have the same population-weighted relaxation rates and thus the same linewidths, which is significantly larger than the pure free state line width. In this limit a final peak intensity of 25% is expected. Given the fact that the observed peak intensity decrease match well with the expectations for the formation of a 67 kDa complex, this indicates that the Ubl45-CD complex behaves as a globular assembly rather than two separate domains flexibly linked by the C-terminal tail.

Legend continues on next page

Supplementary figure 2. Related to Figure 2; Continued

B. Close-up views of the peak trajectories observed for residues that are part of the intermolecular interface in the Ubl45-CD-Ub structure of Rougé et al. The absence of clear CSPs and the absence of peak intensity decrease lower than overall average (see panel C) indicate that Ubl45, in absence of Ub, is not involved in a specific interaction with CD as predicted from the Rougé structure.

C. Simulated NMR peak trajectories (left) and peak intensity ratios (right) as function of chemical shift perturbation and off-rate (k_{off}). The simulated system is based on the Ubl45-CD interaction, using the appropriate molecular weights for the free protein and complex, the experimentally used titration scheme, and the affinity as determined by SPR. The CSP between free and bound state (indicated with a black circle) was systematically varied between 0 and maximally 0.5 ppm for ^{1}H and 2 ppm for ^{15}N. The titration was simulated for three combinations of on- and off-rates as indicated. Extrapolating from the experimentally observed off-rate for the Ubl45–CDUb interaction (K_{D} 1 μM, k_{off} 1 s^{-1}, Figure 3c and S3b), the most likely scenario corresponds to k_{off} 300 s^{-1} and k_{on} $1{\cdot}10^{6}$ s^{-1}, which matches well with diffusion-controlled on-rate for biomolecular systems. At this off-rate, only small CSPs can be observed and residues with large chemical shift changes will show a strong reduction in peak intensity, beyond what is expected based on the amount of free protein. Higher off-rates will allow to observe larger CSPs. For very slow off-rates (< 10 s^{-1}) the free state and bound state become essentially isolated species and the observed peak will no longer encode properties of the bound state. The bound state will have peak intensity corresponding at most to ~10% (60% population of peak broadened 2.5 times in each dimension) of the apo state peak in the limit of no exchange and thus hardly observable in practice because of additional exchange broadening. The experimentally observed peak intensity ratio at position of the *apo* peak (42%) is best reproduced using a k_{off} of 50 s^{-1} (k_{on} $2{\cdot}10^{5}$ s^{-1}). Still, also for this slow exchange a large decrease in peak intensities (down to 10%) is expected for residues with large CSPs. The grey area indicates the expected peak intensities for residues without CSP from the slow to fast exchange limit. Peak intensities at the free state chemical shift position in the apo and 10 equivalent added spectrum are indicated as I_0 and I_{10}, respectively. In cases a CSP can be observed the actual intensity ratio will be higher, at most equal to the zero ppm CSP case; these are marked with a *.

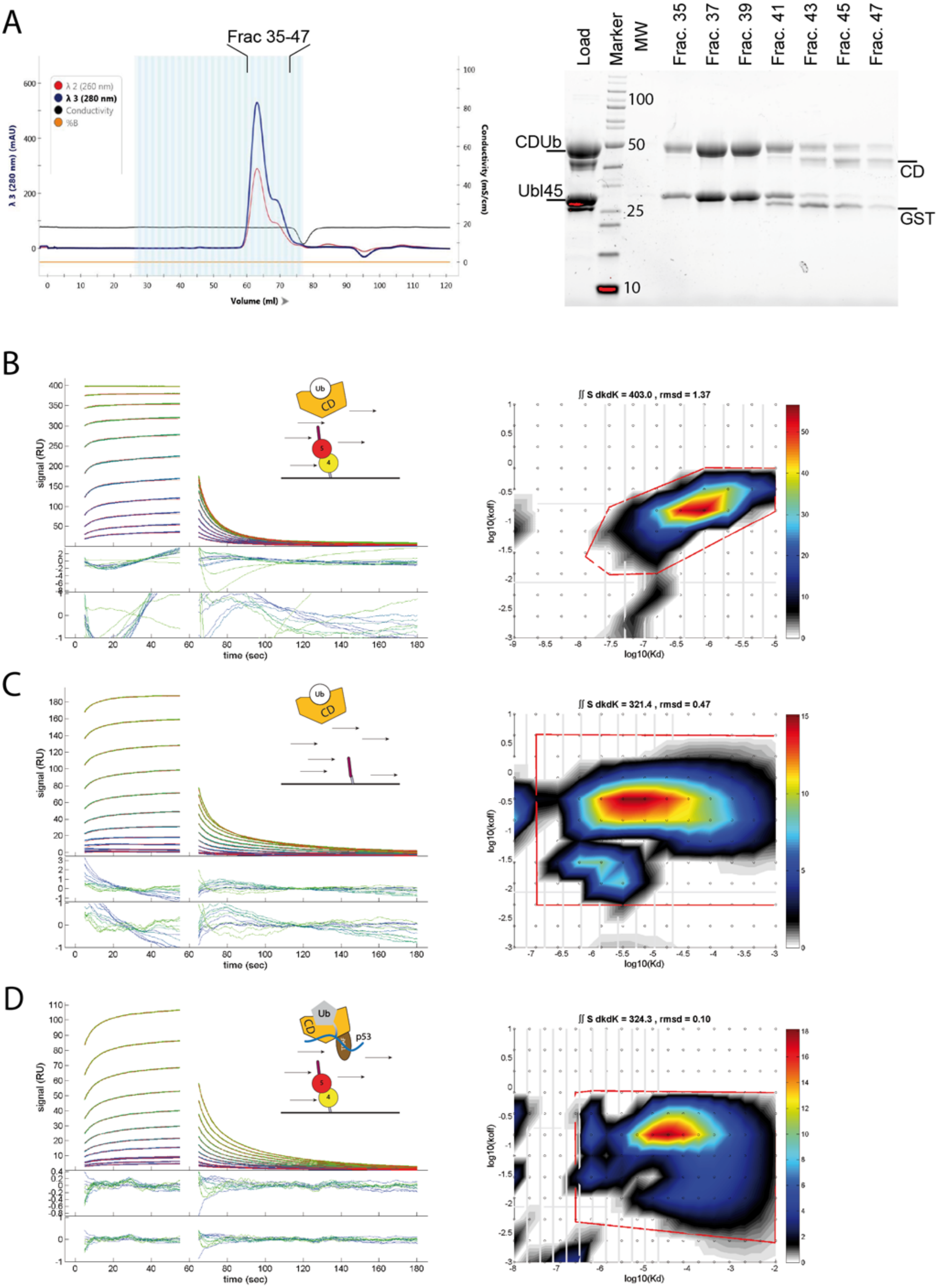

Supplementary figure 3. Related to Figure 3 and Figure 4d;

Supplementary figure 3. Related to Figure 3 and Figure 4d;

A. Co-elution of CDUb and Ubl45 on Superdex75 gel filtration indicates a strong affinity. After incubation of CD and Ub-PA with Ubl45 to generate covalently bound CDUb, the product co-elutes with Ubl45 on gel filtration. The fractions indicated in the filtration profile are run on SDS-PAGE and imaged using the StainFree approach on the imager.
B. The SPR curves from BiaCore were fitted using EvilFit to obtain both K_D and k_{off} values for the interaction between Ubl45 and CDUb. For every fitted interaction, the panel shows the fit of the curves (rainbow-coloured) with the residuals displayed below. The resulting heat map of the fitted values for K_D and k_{off} is shown beside.
C. The SPR curves from the interaction between the C-terminal peptide and CDUb fitted using EvilFit.
D. The raw SPR data of the interaction between TCD-p53Ub and Ubl45 (Figure 4d) fitted using EvilFit.

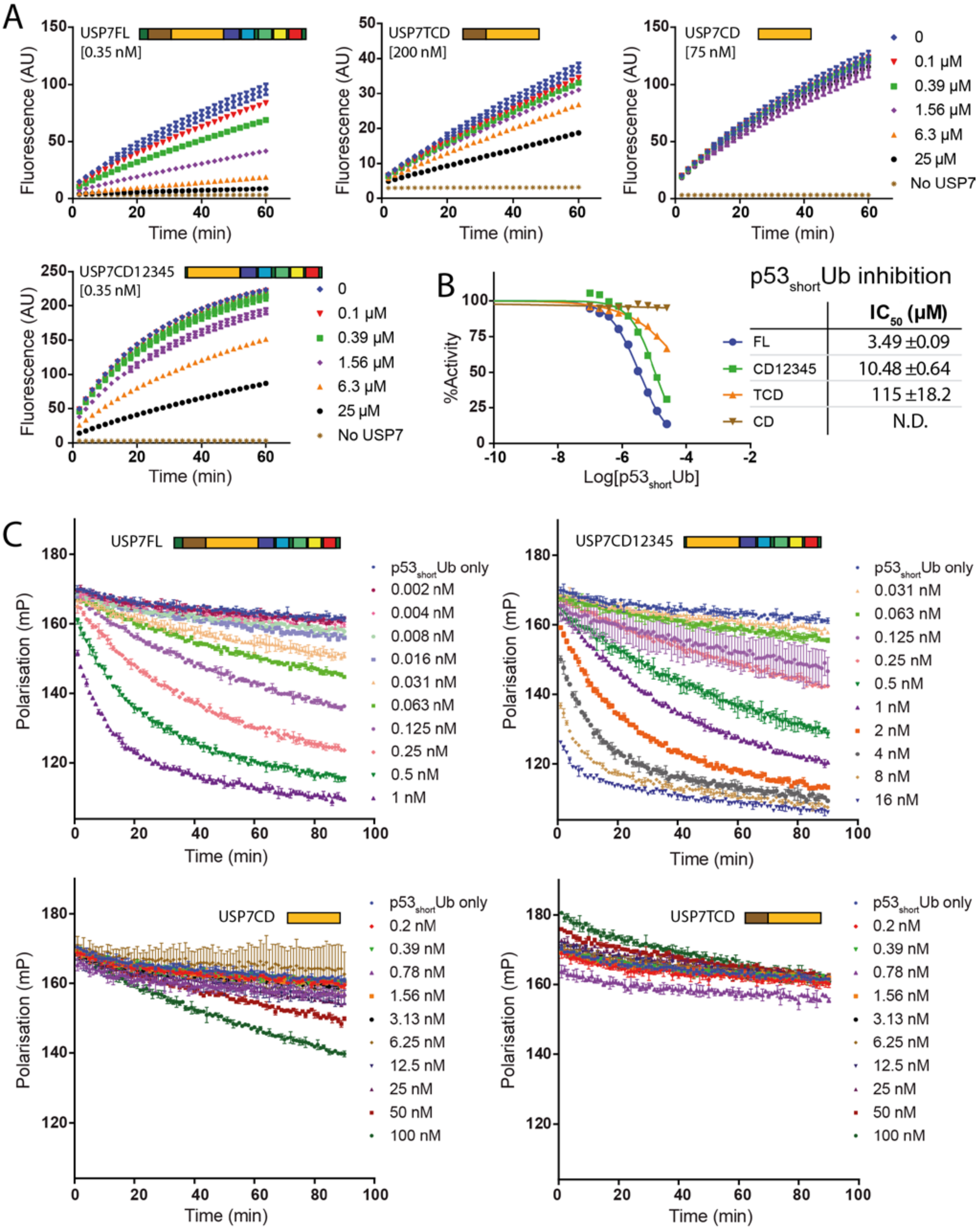

Supplementary figure 4. Related to Figure 5 and Figure 6b;

Supplementary figure 4. Related to Figure 5 and Figure 6b;

A. The inhibition assay (see Figure 5a) was repeated using the short non-hydrolysable p53Ub substrate, showing less inhibition when the TRAF motifs are left out.
B. The initial velocities for the assay in A. are plotted against the used inhibitor concentration, yielding IC_{50}-values as stated in the adjacent table.
C. The FP-assay as done in figure 6b is performed using ^{TAMRA}p53Ub$_{short}$ for the various protein constructs. The used protein concentrations are stated, they differ between constructs to cope with their difference in activity. All data points in A and C are the mean ± SD of at least n=2 experiments. The reported values in B include the SD (±).

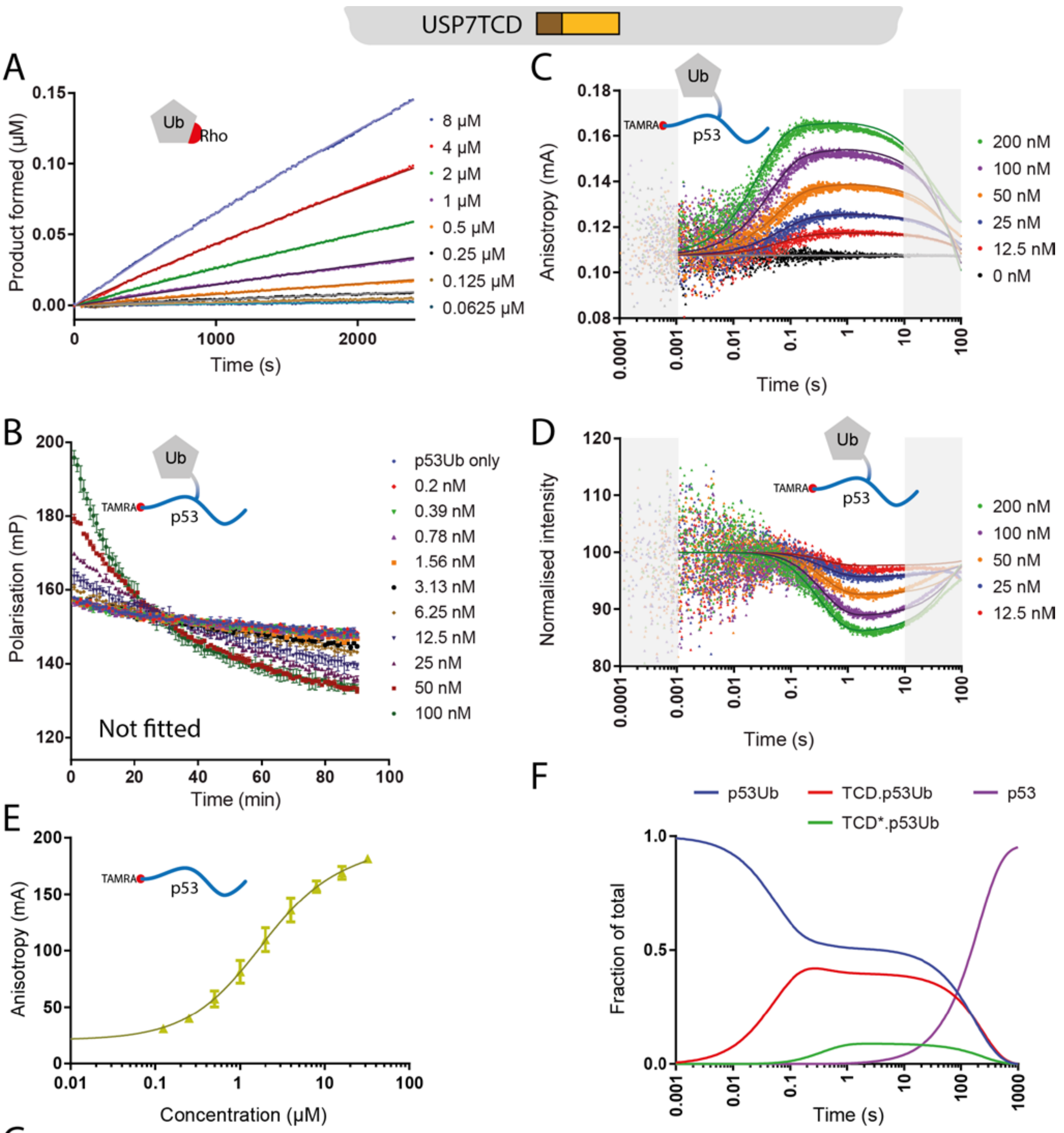

G

		k_f		k_r		Steady state parameters
1 TCD + UbRho	⇋ TCD.UbRho	179$^{\varepsilon}$		2540	±1330	
2 TCD.UbRho	⇋ TCD*.UbRho	0.572$^{\gamma}$	±0.004	2.52$^{\beta}$	±0.018	Ub-Rho: K_M 11.6 μM
4 TCD*.UbRho	⇋ TCD.Ub + Rho	0.0495$^{\delta}$	±0.39	0$^{\theta}$		k_{cat} 0.009 s^{-1}
7 TCD.Ub	⇋ TCD + Ub	40$^{\alpha}$		179$^{\varepsilon}$		k_{cat}/K_M 0.78 $mM^{-1}s^{-1}$
1 TCD + p53Ub	⇋ TCD.p53Ub	179$^{\varepsilon}$		5.82	±0.11	Ub-p53
2 TCD.p53Ub	⇋ TCD*.p53Ub	0.572$^{\gamma}$	±0.004	2.52$^{\beta}$	±0.018	K_M 0.027 μM
4 TCD*.p53Ub	⇋ TCD.p53 + Ub	0.0495$^{\delta}$	±0.39	0$^{\theta}$		k_{cat} 0.009 s^{-1}
6 TCD + p53	⇋ TCD.p53	179$^{\varepsilon}$		160	±11.1	k_{cat}/K_M 338 $mM^{-1}s^{-1}$
7 TCD.Ub	⇋ TCD + Ub	40$^{\alpha}$		179$^{\varepsilon}$		

Supplementary figure 5. Related to Figure 6;

Supplementary figure 5. Related to Figure 6;

Global fitting of TCD data using KinTek is displayed in a similar fashion as figure 6. Icons in each panel indicate the substrate used. Non-fitted are indicated in their panel.

A. Minimal substrate activity assay of TCD (20 nM).
B. FP enzyme activity assay on ^{TAMRA}p53Ub (100 nM) in plate reader setup. The used amounts of TCD are indicated in the legends, the data points are the mean ± SD of n=2 experiments.
C. Stopped flow FP enzyme activity assay on ^{TAMRA}p53Ub (50 nM), showing anisotropy signal allows observation of the binding and hydrolysis phases.
D. The intensity signal for the experiment in C indicates a change in chemical environment upon binding of substrate. All stopped-flow experiment are an addition of n=10 separate measurements.
E. The end-point FP experiment measuring the affinity of TCD for the p53 product (Figure 5c) was used to determine product binding of Step 6.
F. Upon fitting of all experiments described here the fraction of each p53-substrate state could be modelled in an equimolar (1 to 1) setting.
G. The model description used in KinTek to fit all the data. The intermediate state of TCD is indicated with an asterisk (TCD*). The forward and reverse rates reflect the optimal ratio that models the individual steps, since the experiment does not have sufficient resolution to fully resolve rates. The independently determined diffusion rate (ε) and the irreversible reaction (θ) have been fixed, whilst values with identical superscript identifiers (α,β,γ,δ) have been co-refined. The rates are depicted with their respective SD (±).

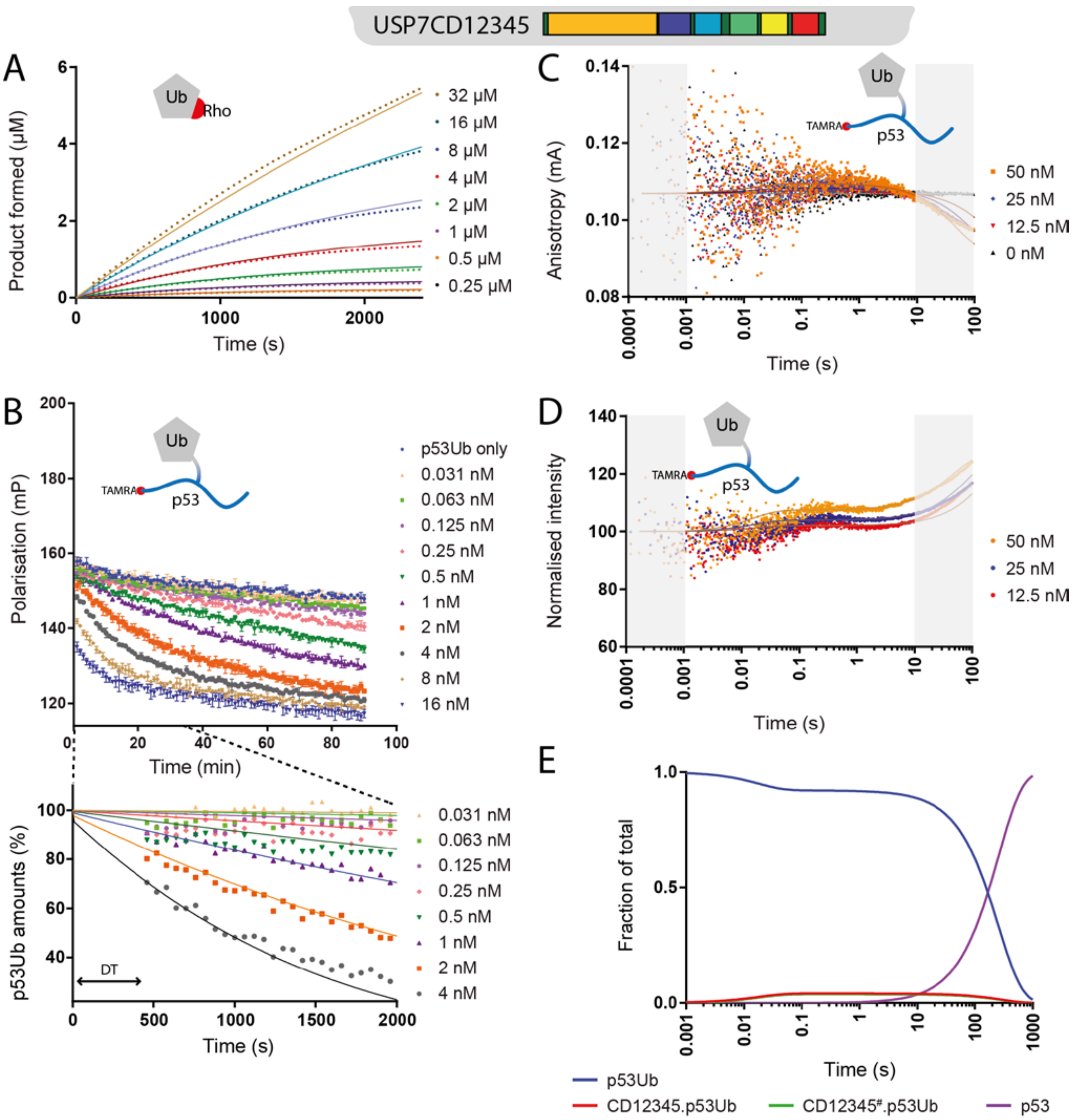

F

	k_f		k_r	
1 CD12345 + UbRho ⇋ CD12345.UbRho	179^{ε}		1870	±1210
3 CD12345.UbRho ⇋ CD12345#.UbRho	800^{γ}	±881	917^{β}	±1100
4 CD12345#.UbRho ⇋ CD12345.Ub + Rho	3.0	±1.3	0^{θ}	
7 CD12345.Ub ⇋ CD12345 + Ub	533^{α}	±887	179^{ε}	
1 CD12345 + p53Ub ⇋ CD12345.p53Ub	179^{ε}		80.7	±21.8
3 CD12345.p53Ub ⇋ CD12345#.p53Ub	800^{γ}	±881	917^{β}	±1103
4 CD12345#.p53Ub ⇋ CD12345.p53 + Ub	0.118	±0.003	0^{θ}	
7 CD12345.Ub ⇋ CD12345 + Ub	533^{α}	±887	179^{ε}	

Steady state parameters

Substrate	K_M	k_{cat}	k_{cat}/K_M
Ub-Rho	5.6 µM	1.40 s^{-1}	250 $mM^{-1}s^{-1}$
Ub-p53	0.24 µM	0.06 s^{-1}	228 $mM^{-1}s^{-1}$

Supplementary figure 6. Related to Figure 6;

Supplementary figure 6. Related to Figure 6;

Global fitting of CD12345 data using KinTek is displayed in a similar fashion as figure 6. Icons in each panel indicate the substrate used.

A. Minimal substrate activity assay of CD12345 (1 nM).
B. FP enzyme activity assay on ^{TAMRA}p53Ub (100 nM) in plate reader setup. The used amounts of CD12345 are indicated in the legends, the data points are the mean ± SD of n=2 experiments.
C. Stopped flow FP enzyme activity assay on ^{TAMRA}p53Ub (50 nM), showing anisotropy signal allows observation of the binding and hydrolysis phases.
D. The intensity signal for the experiment in C indicates a change in chemical environment upon binding of substrate. All stopped-flow experiment are an addition of n=10 separate measurements.
E. Upon fitting of all experiments described here the fraction of each p53-substrate state could be modelled in an equimolar (1 to 1) setting.
F. The model description used in KinTek to fit all the data. The intermediate state of CD12345 is indicated with a pound sign. The independently determined diffusion rate (ε) and the irreversible reaction (θ) were introduced as fixed values, whilst values with identical superscript identifiers (α,β,γ,δ) were co-refined. The rates are depicted with their respective SD (±).

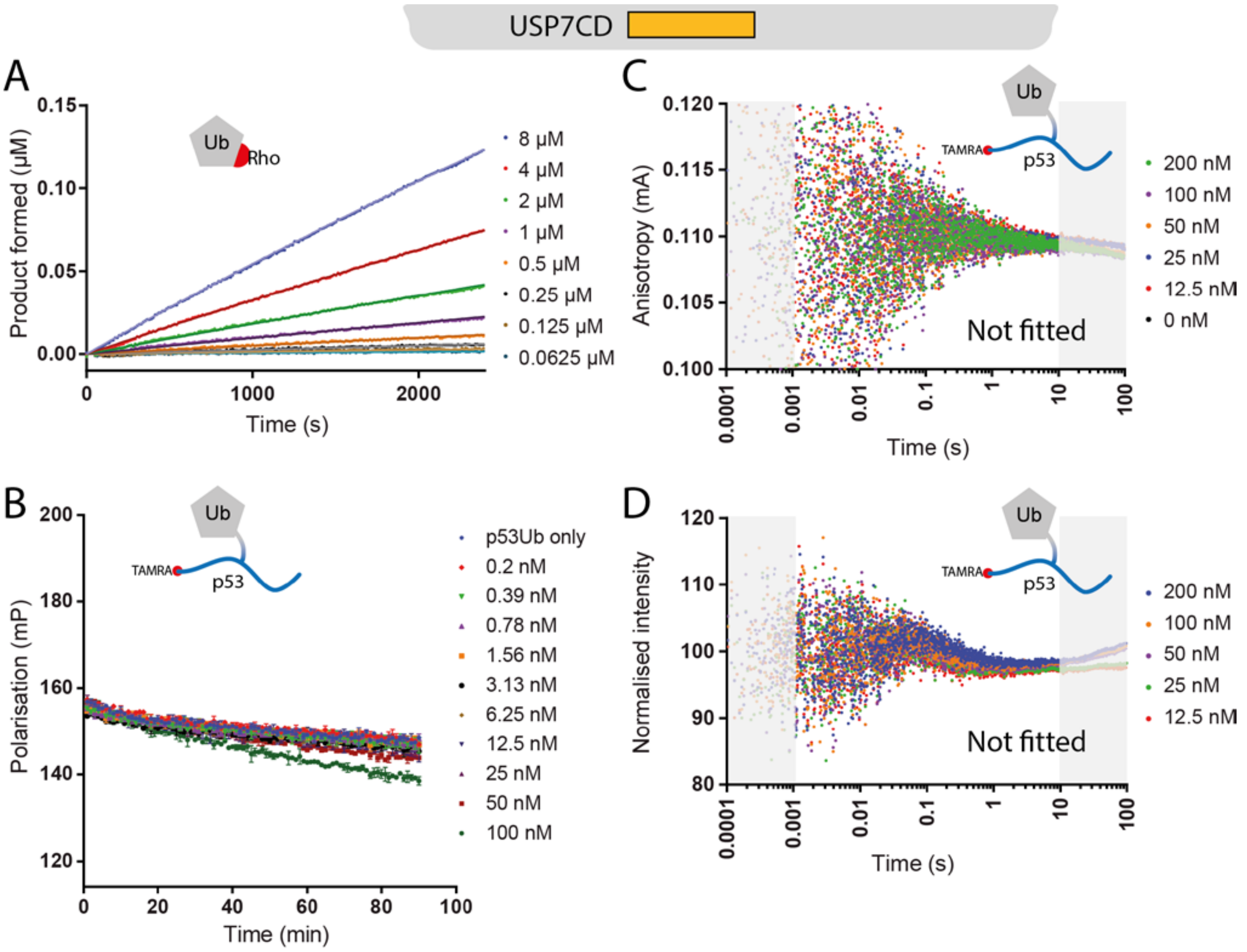

E

	Reaction		k_f		k_r		Steady state parameters
1	CD + UbRho	⇋ CD.UbRho	179[ε]		3330	±734	Ub-Rho: K_M 15.2 μM
2	CD.UbRho	⇋ CD*.UbRho	0.572[β]		2.52[β]		k_{cat} 0.008 s^{-1}
4	CD*.UbRho	⇋ CD.Ub + Rho	0.045	±0.012	0[θ]		k_{cat}/K_M 0.54 $mM^{-1}s^{-1}$
7	CD.Ub	⇋ CD + Ub	71.2[α]	±14.7	179[ε]		
1	CD + p53Ub	⇋ CD.p53Ub	179[ε]		3330	±114	Ub-p53: K_M 15.2 μM
2	CD.p53Ub	⇋ CD*.p53Ub	0.572[β]		2.52[β]		k_{cat} 0.012 s^{-1}
4	CD*.p53Ub	⇋ CD.p53 + Ub	0.064	±0.019	0[θ]		k_{cat}/K_M 0.76 $mM^{-1}s^{-1}$
7	CD.Ub	⇋ CD + Ub	71.2[α]	±14.7	179[ε]		

Supplementary figure 7. Related to Figure 6;

Supplementary figure 7. Related to Figure 6;

Global fitting of CD data using KinTek is displayed in a similar fashion as figure 6. Icons in each panel indicate the substrate used. Non-fitted are indicated in their panel.

A. Minimal substrate activity assay of CD (20 nM).
B. FP enzyme activity assay on ^{TAMRA}p53Ub (100 nM) in plate reader setup. The used amounts of CD are indicated in the legends, the data points are the mean ± SD of n=2 experiments.
C. Stopped flow FP enzyme activity assay on ^{TAMRA}p53Ub (50 nM), showing anisotropy signal allows observation of the binding and hydrolysis phases.
D. The intensity signal for the experiment in C indicates a change in chemical environment upon binding of substrate. All stopped-flow experiment are an addition of n=10 separate measurements.
E. The model description used in KinTek to fit all the data. The intermediate state of CD indicated with an asterisk, is included based on the findings for TCD (Supplementary Figure 4). These rates (β) were fixed in the refinement, just as the determined diffusion rate (ε) and the irreversible hydrolysis reaction (θ). The rates are depicted with their respective SD (±).

A USP7FL

#	Reaction		k_f		k_r	
1	USP7 + UbRho	⇋ USP7.UbRho	179	x	503	c
4	USP7.UbRho	⇋ USP7.Ub + Rho	1.1	c	0	x
7	USP7.Ub	⇋ USP7 + Ub	557	x	179	x
1	USP7 + p53Ub	⇋ USP7.p53Ub	179	x	16.6	b
2	USP7.p53Ub	⇋ USP7*.p53Ub	7.97	b	763	b
3	USP7*.p53Ub	⇋ USP7#.p53Ub	60.4	d	0.02	d
4	USP7#.p53Ub	⇋ USP7*.p53 + Ub	1.1	c	0	x
5	USP7*.p53	⇋ USP7.p53	7.97	b	8.03	o
6	USP7 + p53	⇋ USP7.p53	179	x	34.4	x
7	USP7.Ub	⇋ USP7 + Ub	557	x	179	x

B

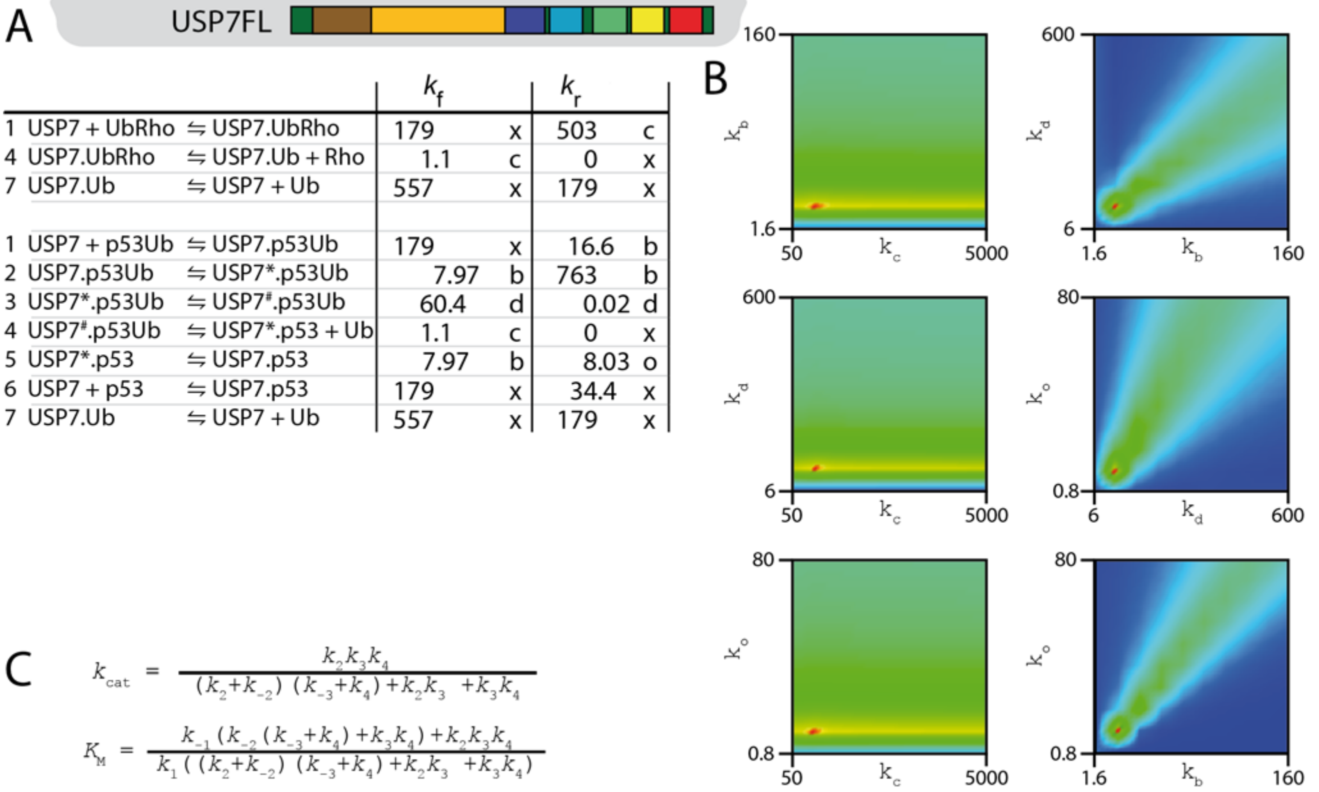

C

$$k_{cat} = \frac{k_2 k_3 k_4}{(k_2+k_{-2})(k_{-3}+k_4)+k_2k_3 \quad +k_3k_4}$$

$$K_M = \frac{k_{-1}(k_{-2}(k_{-3}+k_4)+k_3k_4)+k_2k_3k_4}{k_1((k_2+k_{-2})(k_{-3}+k_4)+k_2k_3 \quad +k_3k_4)}$$

$$\mathrm{USP7} \underset{k_{-1}}{\overset{k_{+1},\ \mathrm{p53Ub}}{\rightleftharpoons}} \mathrm{USP7.p53Ub} \underset{k_{-2}}{\overset{k_{+2}}{\rightleftharpoons}} \mathrm{USP7^{*}.p53Ub} \underset{k_{-3}}{\overset{k_{+3}}{\rightleftharpoons}} \mathrm{USP7^{\#}.p53Ub} \underset{k_{-4}}{\overset{k_{+4},\ \mathrm{Ub}}{\rightleftharpoons}} \mathrm{USP7^{*}.p53}$$

D USP7TCD

#	Reaction		k_f		k_r	
1	TCD + UbRho	⇋ TCD.UbRho	179	x	2536	c
2	TCD.UbRho	⇋ TCD*.UbRho	0.572	c	2.52	b
4	TCD*.UbRho	⇋ TCD.Ub + Rho	0.0495	d	0	d
7	TCD.Ub	⇋ TCD + Ub	40	x	179	d
1	TCD + p53Ub	⇋ TCD.p53Ub	179	x	5.82	o
2	TCD.p53Ub	⇋ TCD*.p53Ub	0.572	c	2.52	b
4	TCD*.p53Ub	⇋ TCD.p53 + Ub	0.0495	d	0	x
6	TCD + p53	⇋ TCD.p53	179	x	160	d
7	TCD.Ub	⇋ TCD + Ub	40	x	179	x

E

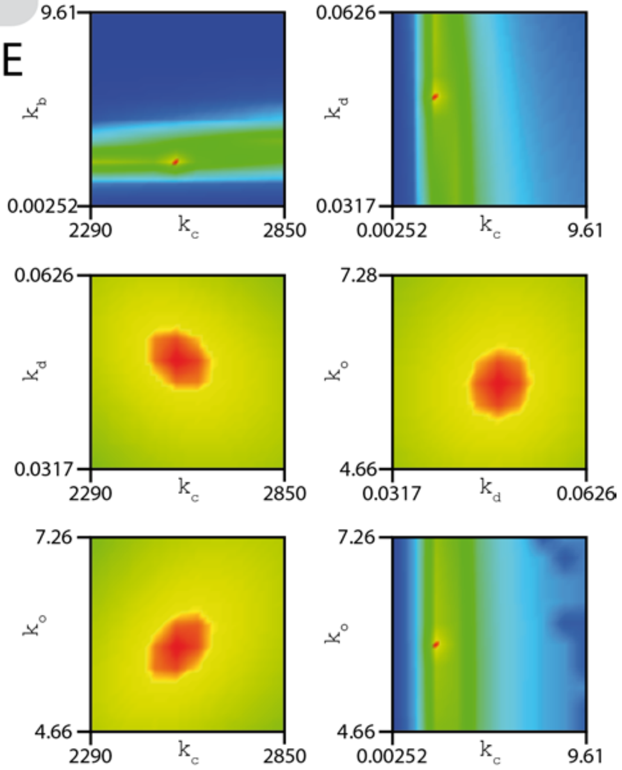

F

$$k_{cat} = \frac{k_3 k_4}{k_3 + k_{-3} + k_4}$$

$$K_M = \frac{k_{-1}(k_{-3}+k_4) \quad +k_3k_4}{k_1(k_3+k_{-3}+k_4)}$$

$$\mathrm{TCD} \underset{k_{-1}}{\overset{k_{+1},\ \mathrm{p53Ub}}{\rightleftharpoons}} \mathrm{TCD.p53Ub} \underset{k_{-3}}{\overset{k_{+3}}{\rightleftharpoons}} \mathrm{TCD^{*}.p53Ub} \underset{k_{-4}}{\overset{k_{+4},\ \mathrm{Ub}}{\rightleftharpoons}} \mathrm{TCD.p53}$$

Supplementary figure 8. Related to Figure 6 and Supplementary figure 5;

Supplementary figure 8. Related to Figure 6 and Supplementary figure 5;

A. The statistical analyses as performed by the FitSpace module of KinTek. The equation constants were linked were applicable and labelled (b,c,d or o). Constants that are marked with an x were locked during fitting and analysis of USP7 FL.
B. The statistical analyses by KinTek of these constants are depicted in the heat plots.
C. To calculate steady state parameters from the KinTek fits the depicted formulas are used. The relationship of the used constants is drawn schematically in the simplified reaction scheme.
D. The same analyses as in A. performed for the TCD construct.
E. With the accompanying statistical analyses, performed by KinTek.
F. The calculation of steady state parameters for reactions with one intermediate state requires the formulas depicted.

Supplementary references

1. Shen, Y. & Bax, A. Protein backbone and sidechain torsion angles predicted from NMR chemical shifts using artificial neural networks. *J. Biomol. NMR* **56,** 227–241 (2013).
2. Li, A., Ziehr, J. L. & Johnson, K. A. A new general method for simultaneous fitting of temperature and concentration dependence of reaction rates yields kinetic and thermodynamic parameters for HIV reverse transcriptase specificity. *J. Biol. Chem.* **292,** 6695–6702 (2017).
3. Toseland, C. P. Fluorescence to Study the ATPase Mechanism of Motor Proteins. in 67–86 (Springer, Basel, 2014). doi:10.1007/978-3-0348-0856-9_4
4. Johnson, K. A. Chapter 23 Fitting Enzyme Kinetic Data with KinTek Global Kinetic Explorer. in *Methods in enzymology* **467,** 601–626 (2009).
5. Johnson, K. A., Simpson, Z. B. & Blom, T. FitSpace explorer: an algorithm to evaluate multidimensional parameter space in fitting kinetic data. *Anal. Biochem.* **387,** 30–41 (2009).
6. Kabsch, W. & Sander, C. Dictionary of protein secondary structure: Pattern recognition of hydrogen-bonded and geometrical features. *Biopolymers* **22,** 2577–2637 (1983).
7. Helgstrand, M., Härd, T. & Allard, P. Simulations of NMR pulse sequences during equilibrium and non-equilibrium chemical exchange. *J. Biomol. NMR* **18,** 49–63 (2000).
8. Allard, P., Helgstrand, M. & Härd, T. The Complete Homogeneous Master Equation for a Heteronuclear Two-Spin System in the Basis of Cartesian Product Operators. *J. Magn. Reson.* **134,** 7–16 (1998).
